# The Influence of Host and Bacterial Genotype on the Development of Disseminated Disease with *Mycobacterium tuberculosis*

**DOI:** 10.1371/journal.ppat.1000034

**Published:** 2008-03-28

**Authors:** Maxine Caws, Guy Thwaites, Sarah Dunstan, Thomas R. Hawn, Nguyen Thi Ngoc Lan, Nguyen Thuy Thuong Thuong, Kasia Stepniewska, Mai Nguyet Thu Huyen, Nguyen Duc Bang, Tran Huu Loc, Sebastien Gagneux, Dick van Soolingen, Kristin Kremer, Marianne van der Sande, Peter Small, Phan Thi Hoang Anh, Nguyen Tran Chinh, Hoang Thi Quy, Nguyen Thi Hong Duyen, Dau Quang Tho, Nguyen T. Hieu, Estee Torok, Tran Tinh Hien, Nguyen Huy Dung, Nguyen Thi Quynh Nhu, Phan Minh Duy, Nguyen van Vinh Chau, Jeremy Farrar

**Affiliations:** 1 Oxford University Clinical Research Unit, Hospital for Tropical Diseases, District 5, Ho Chi Minh City, Vietnam; 2 Centre for Clinical Vaccinology and Tropical Medicine, Churchill Hospital, Old Road, Headington, Oxford, United Kingdom; 3 Centre for Molecular Microbiology and Infection, Imperial College, London, United Kingdom; 4 University of Washington School of Medicine, Seattle, Washington, United States of America; 5 Institute for Systems Biology, Seattle, Washington, United States of America; 6 The Hospital for Tropical Diseases, District 5, Ho Chi Minh City, Vietnam; 7 Pham Ngoc Thach Hospital for Tuberculosis and Lung Diseases, District 5, Ho Chi Minh City, Vietnam; 8 Tuberculosis Reference Laboratory, National Institute for Public Health, Bilthoeven, The Netherlands; 9 Bill and Melinda Gates Foundation, Seattle, Washington, United States of America; 10 Hung Vuong Obstetric Hospital, Hung Vuong Street, Ho Chi Minh City, Vietnam; Johns Hopkins School of Medicine, United States of America

## Abstract

The factors that govern the development of tuberculosis disease are incompletely understood. We hypothesized that some strains of *Mycobacterium tuberculosis* (*M. tuberculosis*) are more capable of causing disseminated disease than others and may be associated with polymorphisms in host genes responsible for the innate immune response to infection. We compared the host and bacterial genotype in 187 Vietnamese adults with tuberculous meningitis (TBM) and 237 Vietnamese adults with uncomplicated pulmonary tuberculosis. The host genotype of tuberculosis cases was also compared with the genotype of 392 cord blood controls from the same population. Isolates of *M. tuberculosis* were genotyped by large sequence polymorphisms. The hosts were defined by polymorphisms in genes encoding Toll-interleukin 1 receptor domain containing adaptor protein (*TIRAP*) and Toll-like receptor-2 (*TLR*-2). We found a significant protective association between the Euro-American lineage of *M. tuberculosis* and pulmonary rather than meningeal tuberculosis (Odds ratio (OR) for causing TBM 0.395, 95% confidence intervals (C.I.) 0.193–0.806, P = 0.009), suggesting these strains are less capable of extra-pulmonary dissemination than others in the study population. We also found that individuals with the C allele of *TLR*-2 T597C allele were more likely to have tuberculosis caused by the East-Asian/Beijing genotype (OR = 1.57 [95% C.I. 1.15–2.15]) than other individuals. The study provides evidence that *M. tuberculosis* genotype influences clinical disease phenotype and demonstrates, for the first time, a significant interaction between host and bacterial genotypes and the development of tuberculosis.

## Author Summary

Tuberculosis, caused by the bacterium *Mycobacterium tuberculosis*, kills over 2 million people each year. It is estimated that approximately one-third of the world population is infected with *M. tuberculosis*, though the majority will never develop active disease. The most severe form of tuberculosis occurs when the bacterium spreads to the brain to cause meningitis. We examined whether the genetic variation of the person and the bacteria influenced the type of disease a person develops. We have previously shown that certain mutations in genes of the human immune system can predispose adults in Vietnam to developing tuberculous meningitis. In this study we show that some strains of *M. tuberculosis* commonly found in Europe and America are less likely to cause tuberculous meningitis in Vietnamese adults than strains predominantly found in Asia. We then looked at the interaction between *M. tuberculosis* strains and mutations in human immune genes and show that a particular mutation, TLR2 T597C, is more commonly found in patients infected with the East-Asian/Beijing strains of *M. tuberculosis*. This is the first study to look at both the host and pathogen genotypes together in tuberculosis infection, and the findings suggest that the outcome of exposure to *M. tuberculosis* can depend on both the human genotype and the bacterial genotype.

## Introduction

It is estimated that one third of the world's population is infected with *Mycobacterium tuberculosis* (*M. tuberculosis*), although the majority will never develop active disease. The factors that govern the development of tuberculosis disease are complex and incompletely understood. Various factors have been clearly associated with increased susceptibility to tuberculosis. HIV infection is by far the most important; it increases the lifetime risk of sub-clinical infection converting to active disease from 1 in 10 to 1 in 3 [1] and is strongly associated with disseminated disease. Defining the contribution of host genetic polymorphisms to disease susceptibility has been more difficult. Studies have suggested polymorphisms in several genes are associated with the development of pulmonary tuberculosis. Some of the genes with polymorphisms that have been validated in multiple studies and may have an effect on gene function include solute carrier family 11, member 1 (SLC11A1, formerly NRAMP1) [2]–[6], interferon gamma [7],[8], *TIRAP*/MAL [9], P2XA7 [10],[11], and CCL2 (or MCP-1), [12]–[14]. Others have shown the less common extra-pulmonary manifestations of tuberculosis may have a different host genetic susceptibility profile and have implicated various polymorphism in components of the innate host response to infection [15] [16],[17] [18],[19]. We have recently reported associations between the development of TBM and single nucleotide polymorphisms (SNP) in the Toll-interleukin-1 receptor domain containing adaptor protein (*TIRAP*) and Toll-like receptor-2 (*TLR*-2) genes [19],[20]. However, tuberculosis disease results from the interactions between host and bacteria and there have been no studies examining the influence and relationship of both host and bacterial genotype variation on clinical disease phenotype.

**Figure 1:**
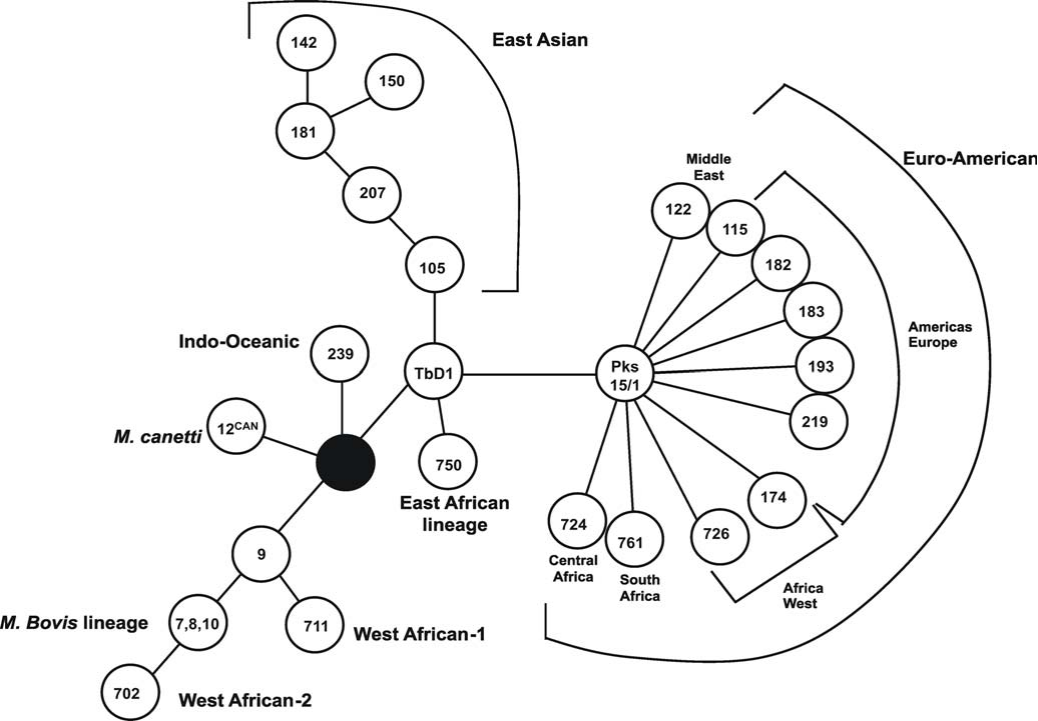
*Mycobacterium tuberculosis* lineages defined by large sequence polymorphism (LSP) analysis. The circles represent Region of difference (RD region) deleted in each lineage. Only East Asian, Indo-Oceanic and Euro-American lineages were identified in Vietnam.

*M. tuberculosis* exhibits a clonal population structure [21],[22] and therefore was regarded until recently as an organism with little relevant genetic variation [23]. However, studies examining *M. tuberculosis* isolates from wider geographic distributions using whole genome scanning approaches have revealed a cladal phylogeographic distribution with significant variation between major lineages, each of which is associated with specific geographic regions [24],[25] (Figure 1). The degree to which this genetic variation influences disease phenotype has been difficult to study. *In vitro* and *in vivo* models of infection have shown different genotypes of *M. tuberculosis* induce different patterns of host immune response [26]–[30], but the relevance of these findings to human disease remains uncertain. Epidemiological studies have found some genotypes may be associated with different disease phenotypes. For example, several studies have suggested an association between mycobacterial *plc* gene polymorphism and disseminated extra-pulmonary disease [31]–[33], but these studies have been small, retrospective, or unable to determine if differences are due to host genetic susceptibility or bacterial genetic virulence determinants.

There has been much interest in the Beijing genotype of *M. tuberculosis*, which is highly prevalent in Asia and the states of the former USSR and has been responsible for outbreaks of multi-drug resistant tuberculosis in the USA [23],[34]. Animal models of infection with this genotype have suggested it leads to a hypervirulent phenotype compared with other common strains of *M. tuberculosis* [35]. This behaviour has been attributed to an intact polyketide synthase (*pks 15/1)* gene and the production of a phenolic glycolipid (PGL) [29]. PGL synthesis appears to attenuate the early host immune response to infection and is associated with reduced production of inflammatory cytokines (30). The ability of Beijing strains to elude the host innate immune response may explain why a recent study has found this genotype is associated with haematogenously disseminated disease [36]. Animal infection models suggest haematogenous dissemination of infection occurs before the onset of T-cell mediated immunity [37] and supports the hypothesis that the ability of different strains of *M. tuberculosis* to produce different clinical phenotypes varies dependent upon their interaction with the host innate immune response.

The study described here examined the relationship between polymorphisms in genes responsible for host innate immunity, bacterial genotype, and the development of pulmonary or meningeal tuberculosis. TBM represents the most severe form of haematogenously disseminated tuberculosis causing death or severe disability in more than half of sufferers [38]. We demonstrate that bacterial genotype does influence disease phenotype and interactions between bacterial and host genotype further influence disease expression.

## Results

### Association between bacterial genotype and disease phenotype

#### Spoligotyping, RFLP, and MIRU typing

To investigate whether different strains of *M. tuberculosis* are associated with disseminated disease, we examined isolates from HIV-negative adult patients in Vietnam who either had meningeal disease (n = 187) or localized pulmonary TB (n = 237). Isolates of *M. tuberculosis* were collected from the CSF of patients with meningitis or the sputum of those with pulmonary TB. The median age of TBM patients was 32 years (range 15–78 years) and of pulmonary patients 36 (range 15–89) (Table 1). We then genotyped each strain by 3 standard methods: spoligotyping, RFLP, and MIRU typing. Three pulmonary isolates showed evidence of mixed culture by more than one method on repeated occasions (dual bands on LSP typing, dual peaks on MIRU, secondary banding on RFLP, for example) and were therefore excluded from further analysis. It is not known if these cases represent mixed infections or laboratory contamination but it is likely that in a sample of this size some patients would be infected with multiple strains. 234 pulmonary isolates were therefore included in all further analyses.

**Table 1:** Demographic data for cases of TBM and pulmonary tuberculosis recruited to the study.

	TBM (n = 187)		Pulmonary TB (n = 237)	
	male	female	Male	female
**Age group (years)**				
15–25	20	33	32	28
26–35	25	31	35	19
36–45	20	13	34	17
46–55	13	6	18	10
56–65	6	6	11	8
65+	5	9	10	15
**Total**	**89**	**98**	**140**	**97**
**Address of participants** a				
Urban	24	20	31	21
Sub-urban	8	5	14	9
Rural (HCMC surrounds)	4	13	8	17
Rural south-East	23	22	48	22
Rural south West	30	38	39	28
**Total**	**89**	**98**	**140**	**97**

a Defined as the main place of residence on entry to the study. Urban addresses were those within the central districts of Ho Chi Minh City (HCMC); sub-urban addresses were those within the outer districts of HCMC; rural (HCMC surrounds) addresses were in the immediate surrounding rural districts of HCMC; the other rural addresses were defined by whether they were south east or south west of HCMC.

Table 2 summarises how the methods clustered the isolates and their respective ability to discriminate between strains. Overall, 348/421 (82.7%) of isolates clustered by spoligotyping, of which 159/421 (37.8%) were ST1 or the ‘Beijing’ genotype (including variants lacking additional spacers 37–43) and 74/421 (17.6%) belonged to the Vietnam genotype, ST319 [39]. By RFLP, the single largest cluster, the Hanoi genotype [39], was formed by single copy isolates, n = 119/421 (28.3%). MIRU typing clustered 57.7% (n = 243/421) of isolates. The 3 largest clusters were composed of MIRU 233325173533 (n = 28); MIRU 364225223533 (n = 20), MIRU 223325173533 (n = 15). There was no significant difference (P>0.05) between the proportions clustering in the pulmonary and meningeal tuberculosis groups by any of these three methods and no significant associations were found between any cluster and the two disease phenotypes.

**Table 2:** Spoligotype, IS6110 RFLP and MIRU typing for all *M. tuberculosis* isolates in the study.

Typing technique	All isolates clustering (n = 421)	Major clusters	Median cluster size	Hunter-Gaston Discrimination index a			Pulmonary isolates clustering (n = 234)	TBM isolates clustering (n = 187)
				*Pulmonary*	*TBM*	*All*		
Spoligotyping	348 (82.7%)	ST1 (Beijing) (38%)*,	3	0.842	0.798	0.826	179 (76.5%)	144 (77%)
		ST319 (18%)^b^ †						
RFLP	238 (56.5%)	Ha Noi genotype^c^ †(28.3%)	2	0.932	0.908	0.917	121 (51.7)	94 (50.3%)
		zero copy isolates (5%)^d^ †						
MIRU	243 (57.7%)	233325173533 (6.6%)*	2	0.990	0.986	0.988	112 (47.9%)	99 (52.9%)
		223325173533 (3.5%)*						
		364225223533 (4.7%)†						

a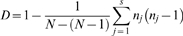 where *N* = the total number of strains in the sample population, *s* = the total number of types described and *n_j_* = the total number of strains belonging to the *j* ^th^ type [52].

b ST319, also known as the Vietnam genotype [39].

c The Ha Noi genotype has a single IS6110 copy and is prevalent throughout Vietnam [53].

d *M. tuberculosis* isolates with no IS6110 insertion elements are relatively common in South-East Asia and have been reported in several studies of Vietnamese strains [53],[54].

*: Isolates of East-Asian Genotype in the LSP typing system of Gagneux et al. [24].

**†:** Isolates of the Indo-Oceanic genotype in the LSP typing scheme of Gagneux et al. [24].

#### LSP typing and the pks 15/17 bp deletion

We next examined whether *M. tuberculosis* clades defined by large-sequence polymorphisms (LSPs) were associated with the clinical disease phenotype. The Indo-oceanic lineage, also known as East-African Indian (EAI) [40], or ancestral lineage [41], with RD239 deleted, represented 104/234 (44.4%) pulmonary isolates and 88/187 (47.1%) of the meningeal isolates (Table 3). The East Asian or ‘Beijing’ lineage (RD105 deleted) represented 87/234 (37.1%) of pulmonary isolates and 81/187 (43.3%) meningeal isolates. There was no significant association between either of these lineages and disease phenotype. However, we found a significant association between the Euro-American lineage and pulmonary rather than meningeal tuberculosis (13% (13/234) v.s 5.9% (7/187), Crude odds ratio for causing TBM 0.40, 95% confidence intervals 0.19–0.80, P = 0.009) (Table 3). We sequenced the *pks* gene codons 54 to 154 to confirm that all isolates in the Euro-American lineage were wild-type, identical to the H37Rv sequence. In addition, we sequenced the *pks* 15/1 gene from 12 isolates randomly selected from the RD105 and RD239 deleted clades and demonstrated all contained the identical 7 bp insertion described in HN878 [35],[42]. As expected, all RD105 or RD239 deleted isolates were subsequently shown to have the *pks* 7 bp insertion by MAS-PCR screening.

**Table 3:** LSP lineages of *M. tuberculosis* isolates causing pulmonary and meningeal tuberculosis.

Group	All isolates (%)	Pulmonary tuberculosis (%)	TBM (%)	χ^2^	P-value	OR [95% CI]^b^
East Asian (RD105 deleted)	168 (39.9)	87 (37.2)	81 (43.3)	1.631	0.20	1.29 [0.87–1.91]
Indo-Oceanic (RD239 deleted)	192 (45.6)	104 (44.4)	88 (47.1)	0.286	0.593	1.11 [0.76–1.63]
Euro-American (pks 15/1 Δ7 bp)	43 (10.2)	32 (13.7)	11 (5.9)	6.88	0.009	0.40 [0.19–0.81]
Undefined a	18 (4.3)	11 (4.7)	7 (3.7)	0.232	0.629	0.79 [0.30–2.08]
Total	421 (100)	234 (100)	187 (100)			

a Undefined isolates failed to generate a product on repeated PCR for one of the two RD regions despite generating product for other PCRs; it is likely these isolates carried additional deletions or mutations in the primer region.

b Odds ratio was calculated comparing the meningeal and pulmonary proportions for each lineage.

To confirm the association was not an artifact of demographic differences between the populations we performed multivariate logistic regression with genotype, disease phenotype, age, sex and the participant address (classified into 5 areas) entered into the model. Age and sex influence susceptibility to extrapulmonary tuberculosis [43], certain genotypes of *M. tuberculosis* are associated with young age in Vietnam [39] and analysis by residential district eliminated any potential bias in urban/rural populations of *M. tuberculosis*. By this analysis the Euro-American isolates were still strongly associated with pulmonary rather than meningeal disease (OR for TBM = 0.40, 95% C.I. 0.20–0.83 P = 0.013).

To provide further support for the biological significance of this finding we investigated whether outcome from TBM was influenced by bacterial lineage. No deaths occurred among those infected with fully drug susceptible Euro-American isolates (n = 0/8), whereas 22.6% (27/119) of patients with susceptible isolates of Indo-Oceanic and East-Asian lineages had died by 9 months (Fisher's exact test, P = 0.201).

### Relationship between host and bacterial genotypes and disease phenotype

The polymorphisms found in the *TIRAP* and *TLR*-2 genes and their associations with disease phenotype have been reported previously [19],[20]. In brief, we found previously that the *TIRAP* SNP C558T and the *TLR*-2 SNP T597C were associated with susceptibility to meningeal rather than pulmonary tuberculosis and this was reconfirmed in the current dataset. Therefore, we examined whether these polymorphisms were associated with infection with any particular bacterial genotype and whether the relationship influenced disease phenotype.

Host genotype was available on 314 patients; *TIRAP* 558 genotype was defined in 313 (145 TBM, 168 pulmonary) and *TLR*2 597 in 306 (141 TBM, 165 pulmonary). The polymorphism frequencies and pathogen genotypes are shown in Table 4. All SNPs were in Hardy Weinberg equilibrium (HWE) in cord-blood control individuals (P≥0.05).

**Table 4:** *TLR*2 T597C SNP allele and bacterial genotype frequencies: comparison with host genotype distribution in the cord blood control group.

Group, lineage	Allele		Genotype			Genotype comparison		Allelic comparison		
	T (frequency)	C (frequency)	TT (frequency)	TC (frequency)	CC (frequency)	χ^2^	P	OR (95% C.I)^a^	χ^2^	P
**Cord blood controls**	564 (0.748)	190 (0.252)	205 (0.544)	154 (0.408)	18 (0.048)			1		
**All isolates**	428 (0.699)	184 (0.301)	153 (0.500)	122 (0.399)	31 (0.101)	7.412	0.025	1.28[1.01–1.62]	4.023	0.045
**Indo-Oceanic**	206 (0.725)	78 (0.275)	76 (0.535)	54 (0.380)	12 (0.085)	2.630	0.268	1.12 [0.83–1.53]	0.553	0.457
**Euro-American**	44 (0.710)	18 (0.290)	16 (0.516)	12 (0.387)	3 (0.097)	1.410	0.494	1.21 [0.69–2.15]	0.443	0.505
**All Indo-Oceanic+Euro-American**	271 (0.728)	101 (0.272)	100 (0.538)	71 (0.382)	15 (0.081)	2.532	0.282	1.11 [0.84–1.47]	0.495	0.481
**East-Asian/Beijing**	157 (0.654)	83 (0.346)	53 (0.442)	51 (0.425)	16 (0.133)	11.635	0.003	1.57 [1.15–2.15	8.048	0.004
**TBM only**										
**All isolates**	187 (0.663)	95 (0.337)	66 (0.468)	55 (0.390)	20 (0.142)	13.596	0.001	1.51 [1.12–2.03]	7.417	0.006
**Indo-Oceanic+Euro-American**	114 (0.704)	48 (0.296)	42 (0.519)	30 (0.370)	9 (0.111)	4.861	0.088	1.25 [0.86–1.82]	1.361	0.243
**East-Asian/Beijing**	73 (0.608)	47 (0.392)	24 (0.400)	25 (0.417)	11 (0.183)	16.390	0.0003	1.91 [1.28–2.86]	10.219	0.001
**Pulmonary isolates**										
**All isolates**	241 (0.730)	89 (0.270)	87 (0.527)	67 (0.406)	11 (0.067)	0.828	0.661	1.10 [0.81–1.47]	0.376	0.539
**Indo-Oceanic+Euro-American**	157 (0.748)	53 (0.252)	58 (0.552)	41 (0.390)	6 (0.057)	0.223	0.895	1.00 [0.71–1.43]	0.0001	0.991
**East-Asian/Beijing**	84 (0.700)	36 (0.300)	29 (0.483)	26 (0.433)	5 (0.083)	1.676	0.433	1.27 [0.82–1.47]	1.244	0.264

a OR was calculated comparing each group to the genotype/allele distribution in the cord blood controls.

We analyzed the distribution of alleles and genotypes of the TB groups in comparison with the cord-blood controls (Table 4). *TIRAP* C558T was associated with susceptibility to TBM as previously reported OR = 2.96 [95% C.I. 1.71–5.11], however, there was no stronger association between *TIRAP* C558T and TB caused by any unique *M. tuberculosis* lineage (data not shown). As previously reported [20], the *TLR*2 T597C polymorphism was associated with all cases of tuberculosis (control vs. all isolates; OR = 1.28 [95% C.I. 1.01–1.62], P = 0.045). However, the allelic association was strongest for TB cases caused by the Beijing genotype isolates (control vs. East Asian/Beijing; OR = 1.57 [95% C.I. 1.45–2.15], P = 0.004).

There was no association between the *TLR*2 597C polymorphism and tuberculosis caused by the Indo-Oceanic (P = 0.457) and Euro-American isolates (P = 0.505).

We next examined whether clinical disease phenotype, pulmonary or meningeal disease, influenced the association between *TLR2* T597C and bacterial genotype. There was no allelic association between TLR2 T597C and pulmonary TB caused by non-Beijing isolates (control vs. pulmonary non-Beijing: OR = 1.00 [95% CI 0.71–1.43] P = 0.991) or for Beijing isolates (control vs. pulmonary East-Asian/Beijing: OR = 1.27 [95% C.I. 0.82–1.47], P = 0.264) (Table 4).

There was an overall association of *TLR*2 T597C with meningeal disease (OR = 1.51 [95% C.I. 1.12–2.03] P = 0.006) but this was not significant for meningeal disease caused by non-Beijing isolates (control vs. TBM non-Beijing OR = 1.25, [95% C.I. 0.86–1.82], P = 0.243). The strongest allelic association was between TLR2 T597C and TBM caused by Beijing genotype isolates (control vs. TBM East Asian/Beijing; OR = 1.91 [95% C.I. = 1.28–2.86], P = 0.001). On genotypic analysis this association was also highly significant (χ^2^ = 16.39, P = 0.0003) (Table 4). We previously used a likelihood ratio test with Bayesian Information Criterion values to determine that the association between *TLR2* T597C genotypes and TB showed best fit with a dominant (comparing 597TT/TC vs. 597CC) rather than a recessive (comparing 597TT vs 597TC/CC) model [20]. When we analyzed the association of TB caused by the Beijing lineage and *TLR2* T597C using a dominant model for all types of clinical TB, we found a highly significant association (Table 5) (control vs. all East Asian/Beijing isolates: OR = 3.07 [95% C.I. 1.51–6.23], P = 0.001]. By comparison, there was no significant association between *TLR2* T597C and TB aused by non-Beijing strains (control vs. all non-Beijing isolates: OR 1.75 (95% CI 0.86–3.56, P = 0.118). The association between *TLR*2 T597C and the Beijing strains was strongest for patients with meningeal TB (control vs. TBM East-Asian Beijing OR = 4.48 [95% C.I. 2.00–10.04], P<0.001). Together, these results suggest that the association of SNP *TLR*2 T597C with TBM is strongest among those infected with the Beijing lineage.

**Table 5:** *TLR*2 T597C genotype comparison between control and tuberculosis groups when the major allele (T) is dominant.

Group, lineage	Dominant model				
	TT+TC (frequency)	CC (frequency)	OR [95% C.I.]^a^	χ^2^	P
**Cord blood controls**	359 (0.952)	18 (0.048)	1		
**All isolates**	275 (0.899)	31 (0.101)	2.25 [1.23–4.10]	7.276	**0.006**
**Indo-Oceanic**	130 (0.915)	12 (0.084)	1.84 [0.86–3.93]	2.560	0.109
**Euro-American**	28 (0.903)	3 (0.097)	2.14 [0.59–7.70]	1.410	0.235
**All Indo-Oceanic+Euro-American**	171 (0.919)	15 (0.081)	1.75 [0.86–3.56]	2.443	0.118
**East-Asian/Beijing**	104 (0.867)	16 (0.133)	3.07 [1.51–6.23]	10.463	**0.001**
**TBM only**					
**All isolates**	121 (0.858)	20 (0.142)	3.30[1.69–6.44]	13.37	**<0.001**
**Indo-Oceanic+Euro-American**	72 (0.153)	9 (0.111)	2.49 [1.08–5.77]	14.826	**0.028**
**East-Asian/Beijing only**	49 (0.817)	11 (0.183)	4.48 [2.00–10.03]	15.359	**<0.001**
**pulmonary isolates**					
**All isolates**	154 (0.933)	11 (0.067)	1.43 [0.66–3.09]	0.811	0.368
**Indo-Oceanic+Euro-American**	99 (0.943)	6 (0.057)	1.21 [0.47–3.13]	0.153	0.695
**East-Asian/Beijing only**	55 (0.916)	5 (0.083)	1.81 [0.65–5.08]	1.315	0.251

a OR was calculated for each group relative to genotype distribution in cord blood controls.

## Discussion

The influence of bacterial and host genotype on the development of different forms of TB has been difficult to study in humans. We have compared bacterial and host genotype, and their interaction, across two large groups of Vietnamese adults with pulmonary or meningeal tuberculosis. The study demonstrated a relationship between *M. tuberculosis* phylogenetic lineage and disease phenotype: disease caused by the Euro-American lineage was significantly more likely to be pulmonary than meningeal, which suggests that this lineage may be less capable of extra-pulmonary dissemination in the study population. However, the proportion of Euro-American isolates in this study population is relatively small and therefore a larger study is required to confirm this finding. It is possible that the predominance of young males among the TBM cases presented a skewed distribution of *M. tuberculosis* lineages or that TBM susceptibility factors differ among the elderly or young children.

It is tempting to speculate that the associations between bacterial lineage and disease phenotype are explained by the presence or absence of a functional *pks* 15/1. Recent studies have suggested that the phenolic glycolipid (PGL) produced by some *pks* 15/1 intact isolates specifically inhibits the innate immune response and may be responsible for a propensity to dissemination [29],[35]. In these studies, production of pro-inflammatory cytokines from *M. tuberculosis*-infected macrophages was inhibited by PGL in a dose-dependent manner. In addition, bacteria producing PGL were more capable of dissemination from the brain to other organs in animal models than others [35]. Isolates unable to express PGL – such as the Euro-American lineages - may conversely cause less extra-pulmonary disease. However, the explanation for our findings is unlikely to be as simple and extrapolation from such model studies is highly speculative. It is becoming increasingly clear that antigenic variation in *M. tuberculosis* is greater than previously thought and the causative mechanism of phenotypic disease variation is unlikely to be a single antigen ‘switch’. PGL synthesis is under complex regulation and cannot be predicted simply by the presence of an intact *pks* 15/1 gene sequence [44].We found no differential association with disease phenotype between the East Asian and Indo-Oceanic Lineages, although it is probable the indo-oceanic isolates do not express the PGL [44].

Of note, the patients infected with Euro-American isolates had lower mortality from TBM compared with patients infected with other lineages. This correlates well with evidence from animal models which showed rabbits infected with these strains had less severe clinical manifestations, milder focal meningeal inflammation and minimal infiltrate despite the presence of significant bacillary loads [35]. The lower mortality in human disease provides further evidence that bacterial genotype may have a significant influence on disease phenotype which could have direct clinical relevance. Bacterial genotyping may allow clinicians to identify those more likely to respond poorly to treatment in which more aggressive treatment might be beneficial. However, the number of TBM patients infected with Euro-American isolates in this study was small and a larger study is required to confirm these findings and examine potential confounders such as BCG vaccination status, immunosuppressive co-morbidities etc.

Recent studies have indicated that the different lineages of *M. tuberculosis* are strongly associated with specific geographical regions [24]. A global phylogeography of *M. tuberculosis* has been proposed which suggests lineages may have become specifically adapted to their populations. Such co-evolution, or its absence, may influence disease expression and indicates interactions between bacterial and host genotype should be studied. We hypothesized that polymorphisms in genes responsible for the innate immune response to infection may influence the host response to infection and may result in increased susceptibility to disease from some bacterial lineages but not others. We found that a polymorphism in the *TLR*2 gene was associated with disease caused by the East Asian or Beijing lineage. This is the first time a relationship between bacterial and host genotype has been observed in TB, although it has previously been observed with other pathogens[45].

*TLR*2 is a trans-membrane protein which recognizes bacterial ligands - such as the 19kDa lipoprotein - and initiates a signal transduction cascade which activates dendritic cells and macrophages. The SNP T597C is a synonymous SNP that is not known to affect gene function, although we have previously demonstrated it was associated with TBM disease severity and the co-existence of miliary tuberculosis, the most extreme form of disseminated tuberculosis [20]. This suggests a polymorphism, or polymorphisms in linkage disequilibrium (LD) with *TLR*2 597C are important in multiple-facets of tuberculosis susceptibility. The causal polymorphism may lie in the promoter region, a regulatory region, or in a nearby gene, and must be identified before its effect on disease pathogenesis and interaction with Beijing genotype strains can be understood. However, it is possible that the causal mutation that is in LD with *TLR2* 597C may be associated with an impaired immune response to *M tuberculosis* and lead to more aggressive disease, prolonged bacteraemia, and an increased chance of seeding to the meninges. The Beijing genotype may further exploit the host susceptibility to infection through its own ability to subvert the host innate immune response. We have previously demonstrated a strong association between Beijing genotype and TBM in HIV positive patients in the same population [46] supporting the hypothesis that infection of an immune suppressed host with an immune subversive bacteria represent a synergistic combination that results in an increased likelihood of disease. There was no overall association of Beijing genotype with TBM in this HIV negative Vietnamese study population, although the proportion of Bejing genotype isolates was greater in the meningeal group (43.3% [81/187] of TBM isolates vs. 37.1% [87/234] pulmonary isolates), this was not significant (P = 0.20). Studies in other ethnicities have shown an association of Beijing genotype with extra-pulmonay disesase [36] and it remains possible that a larger study would show an association too small to reach significance here.

In summary, this study provides evidence that *M. tuberculosis* genotype influences disease phenotype. In addition, although many reports describe host susceptibility or bacterial genetic associations with clinical phenotype in isolation, we have reported the first association between host and bacterial genotype in concert in *M. tuberculosis* disease. Studies of host susceptibility or pathogen virulence should be conducted in the context of both. Future vaccine candidates may need to be evaluated against a range of *M. tuberculosis* genotypes and host ethnicities if they are to prove globally effective, particularly against disseminated disease.

## Methods

This study compared the host and bacterial genotypes of Vietnamese adults with TBM or uncomplicated pulmonary tuberculosis. All patients were from a single ethnicity (Vietnamese Kinh) and were not infected with HIV.

### Disease phenotypes, patient recruitment, and sample collection

The patients were recruited to the study as previously described [19],[20]. Briefly, patients with TBM were recruited at Pham Ngoc Thach Hospital for Tuberculosis and Lung Diseases (PNT) and the Hospital for Tropical Diseases (HTD) in Ho Chi Minh City, Vietnam between March 2000 and April 2003. To enter the study patients had to have clinical evidence of meningitis (nuchal rigidity and abnormal CSF parameters) and *M. tuberculosis* cultured from the CSF, and be >15 years old with a negative HIV test. All patients were followed for 9 months after the start of treatment; disability was assessed in survivors by the modified Rankin score [38].

Adult patients with uncomplicated pulmonary tuberculosis were recruited between September 2003 and December 2004 at 5 district tuberculosis units (DTUs) from Ho Chi Minh City and the surrounding districts, chosen to represent the geographic distribution of isolates among TBM patients in order to avoid an urban/rural bias in one sample set. Cases were defined by the culture of *M. tuberculosis* from sputum, a chest X-ray appearance consistent with active tuberculosis without evidence of miliary or extra-pulmonary tuberculosis, and no clinical evidence of extra-pulmonary disease. As far as possible, patients were prospectively matched to TBM patients by age (+/−5 years) and district of residence, defined in five groups as: urban, sub-urban, rural (surrounding HCMC), rural south-East or rural South-West. Matched patients were recruited from a DTU within each of these districts. Gender matching was attempted but not achieved due to a larger number of men with pulmonary TB attending the DTUs.

The control group comprised of 389 DNA samples extracted from the umbilical cord blood of newborn babies born at Hung Vuong Hospital, Ho Chi Minh City, in 2003. All samples came from unrelated individuals who were ethnic Vietnamese Kinh, as assessed by questionnaire.

Written informed consent was obtained from each patient or an accompanying relative if the patient could not provide consent. All protocols were approved by ethical review committees at the HTD, PNT Hospital for Tuberculosis and Lung Disease, Hung Vuong Hospital and Health Services of Ho Chi Minh City in Vietnam. Ethical approval was also granted by Oxfordshire Clinical Research Ethics Committee UK, Oxford Tropical Research Ethics Committee UK, The University of Washington USA and the Western Institutional Review Board USA.

### Host genotyping

Host genotyping and identification of *TLR*2 and *TIRAP* SNPs have been reported in detail previously [19],[20]. Briefly, polymorphisms in both genes were identified by sequencing a randomly selected sub-group of patients with TBM. All subjects were then genotyped for the designated SNPs by an allele-specific primer extension assay (MassARRAY™, Sequenom, San Diego, USA).

#### *M. tuberculosis* genotyping

All *M. tuberculosis* isolates were genotyped by four established methods: IS*6110* restriction fragment length polymorphisms (RFLP) [47], spacer oligo-nucleotide typing (spoligotyping) [48], 12 allele mycobacterial interspersed repetitive unit (MIRU) typing [49], and large sequence polymorphisms (LSP) defined by deligotyping [50]. RFLP has limited discrimination in low-copy number isolates (<5 IS6110 copies) which are prevalent in Vietnam, spoligotyping is unable to discriminate Beijing genotype isolates, which account for approximately 40% of *M. tuberculosis* isolates in this region, and the discriminatory power of MIRU typing was unknown in Vietnam. LSP typing is a relatively new genotyping technique which has been shown to classify isolates in geographically-related clades.

Briefly, bacterial DNA was extracted from cultures on Lowenstein-Jensen media by cetyl trimethylammonium bromide (CTAB) method [51] and diluted to a working concentration of 15 ng/ml. Spoligotyping [48] and RFLP [47] were carried out according to the standard protocols. MIRU was performed following the method of Supply *et al.* with minor modifications for a Beckman CEQ8000 sequencer [49]. Wellred Oligos were provided by Proligo, Singapore with Dye D2 labelling replacing FAM, dye D3 labelling replacing HEX, and dye D4 labelling replacing NED. Mapmarker 600–1200 bp standard labelled with D1 dye (Bioventures Inc, USA) was included with each run. Assignment of amplicon size was performed manually with reference to the standard.

LSPs were defined following the method of Tsolaki *et al.* [50]. Isolates were first characterised for RD105 and RD239 deletion as it was anticipated that the majority of isolates would contain one of these two deletions. Isolates without RD105 or RD239 were sequenced in the *pks* gene to identify the Euro-American lineage using primers *pks*i GCAGGCGATGCGTCATGGGG and *pks*j TCTTGCCCACCGACCCTGGC to amplify a 520 bp fragment [42].

MAS-PCR was used to screen for pks 15/17 bp deletion with outer primers pks1i 3′-GCAGGCGATGCGTCATGGGG-5′ and pks1j 3′-TCTTGCCCACCGACCCTGGC-5′ [42] and an internal primer pks1insR 3′-ACGGCTGCGGCTCCCGATGCT-5′. The PCR mix contained 0.1 µM each outer primer, 0.2 µM pks1insR, 0.2 mM dNTPs, 1.5 mM MgCl_2_, Hotstart Taq (Qiagen), 1× buffer (supplied with enzyme), 10.85 µl ELGA water and 15 ng DNA template in a final volume of 20 µl. The PCR programme was an initial denaturing of 95°C for 15 minutes, followed by 30 cycles of 94°C for 30 seconds, 67°C for 30 seconds and 72°C for 30 seconds, with a final extension of two minutes at 72°C. Isolates with a 7 bp deletion produced 2 bands of 520 bp and 259 bp while isolates without the deletion produce a single band of 520 bp, validated by comparison with sequencing data for 43 wild-type and 12 Δ7 bp pks15/1 isolates.

### Statistical analysis

Analysis was performed with Bionumerics software (Applied Maths, Sint-Martens Latern, Belgium) and STATA 8 (Texas, USA).

Spoligotyping neighbour joining phylogenetic trees were created with eucldian distance coefficient on Bionumerics software. RFLP phylogenetic trees were created with 2% position tolerance and 1% optimization using Unweighted Pair Group Analysis (UPGMA), dice coefficient on Bionumerics software. MIRU trees were created using UPGMA, categorical multistate coefficient. For all methods, isolates were considered clustered if 100% similarity was observed.

The prevalence of genotypes among meningeal and pulmonary isolates was compared by Chi-square test. The association of LSP genotype and disease phenotype was further analysed by forward stepwise logistic regression model (P of <0.05 to enter; P of >0.055 to remove) to identify variables associated with disease phenotype on multivariate analysis. The variables examined in the model were LSP genotype, site of TB, age, sex and residential district. For analysis of host polymorphisms, allelic and genotypic frequencies were compared between the groups using a Chi square test. We also analyzed the data with recessive and dominant models as previously described [20]. P values of ≤0.05 were considered statistically significant.
